# BMC Medical Ethics reviewer acknowledgement 2015

**DOI:** 10.1186/s12910-016-0093-5

**Published:** 2016-02-09

**Authors:** Clare Partridge

**Affiliations:** BioMed Central, Floor 6, 236 Gray’s Inn Road, London, WC1X 8HB UK

## Abstract

The editors of BMC Medical Ethics would like to thank all our reviewers who have contributed to the journal in Volume 16 (2015).

**Beyene Ademe**

Ethiopia

**Kaoruko Aita**

Japan

**Aslihan Akpinar**

Turkey

**Eman Al Gaai**

Saudi Arabia

**Ghiath Alahmad**

Saudi Arabia

**Afshan Ali**

Saudi Arabia

**James A Anderson**

Canada

**Armand Antommaria**

USA

**John Appleby**

UK

**Kavita Shah Arora**

USA

**Atsushi Asai**

Japan

**Richard Ashcroft**

UK

**Marc Avey**

Canada

**Rahime Aydin Er**

Turkey

**Gabriele Badano**

UK

**Kristine Bærøe**

Norway

**Michael Balboni**

USA

**Francis Barchi**

USA

**Jo Barnes**

New Zealand

**Bruce Barron**

USA

**Steve Bellan**

USA

**Solomon (Solly) Benatar**

South Africa

**Rebecca Bennett**

UK

**Hazel Biggs**

UK

**Giles Birchley**

UK

**Isra Black**

UK

**Jonathan Boote**

UK

**Kirstin Borgerson**

Canada

**Elaine Boyle**

UK

**Iain Brassington**

UK

**Anders Bremer**

Sweden

**Berit Bringedal**

Norway

**Arianne Brinkman-Stoppelenburg**

Netherlands

**Rebecca Brown**

UK

**Teneille Brown**

USA

**Susan Bull**

UK

**Emma Bullock**

Hungary

**Luciana Caenazzo**

Italy

**Megan Campbell**

South Africa

**Emma Cave**

UK

**David Chambers**

USA

**Tracey Chantler**

UK

**Phaikyeong Cheah**

Thailand

**Y.Y. Brandon Chen**

Canada

**Yen-Yuan Chen**

Taiwan

**Jeannie Cimiotti**

USA

**John Coggon**

UK

**Gilberto Corbellini**

Italy

**Amy Corneli**

USA

**Anselm Crombach**

Germany

**Andrew Crowden**

Australia

**Sylvia Cruess**

Canada

**Luis Gabriel Cuervo**

USA

**Barbara Daly**

USA

**Myriam Deveugele**

Belgium

**Veljko Dubljevic**

Canada

**Marleen Eijkholt**

USA

**Carolyn Ells**

Canada

**Jakob Elster**

Norway

**Nathan Emmerich**

UK

**Stefan Eriksson**

Sweden

**Anna Falcó-Pegueroles**

Spain

**Zaher Fanari**

USA

**Anne-Maree Farrell**

Australia

**Michael Fay**

UK

**Richard Fielding**

Hong Kong

**Kjersti Fjørtoft**

Norway

**Paul Ford**

USA

**Nikolaus Forgo**

Germany

**Veronique Fournier**

France

**Sara Fovargue**

UK

**Allessandra Gasparetto**

Italy

**Joel Geiderman**

USA

**Eric Geijteman**

Netherlands

**Paul Wenzel Geissler**

Norway

**Davina Ghersi**

Australia

**David Gibson**

UK

**Noor Giesbertz**

Netherlands

**Simona Giordano**

UK

**Nada Gligorov**

USA

**Trudy Goodenough**

UK

**Christine Grady**

USA

**Gilly Griffin**

Canada

**Danielle Griffiths**

UK

**Marilys Guillemin**

Australia

**Freedom Nkhululeko Gumedze**

South Africa

**Karen Gutierrez**

USA

**Akiko Hagimoto**

Japan

**Richard Hain**

UK

**Daniel Hall**

USA

**Sumaya Hammami**

USA

**Thor Willy Ruud Hansen**

Norway

**Lawrence Hansen**

USA

**Bhakti Hansoti**

USA

**Lena Hedén**

Sweden

**Irma Hein**

Netherlands

**Gert Helgesson**

Sweden

**Matthew Herder**

Canada

**Elizabeth Hill**

UK

**Ming-Jung Ho**

Taiwan

**Bjorn Hofmann**

Norway

**Anna Höglund**

Sweden

**Ruth Horn**

UK

**Samia Hurst**

Switzerland

**Adam Hutchings**

UK

**Dawn Ionescu**

USA

**Jonathan Ives**

UK

**Nichon Jansen**

Netherlands

**Rien Janssens**

Netherlands

**Steven Joffe**

USA

**Franz Johnson**

USA

**Niklas Juth**

Sweden

**Jaranit Kaewkungwal**

Thailand

**Michael Kalichman**

USA

**Dorcas Kamuya**

Kenya

**Daniel Kim**

USA

**Jonathan Kimmelman**

Canada

**Celia Kitzinger**

UK

**Satoshi Kodama**

Japan

**Erwin Kompanje**

Netherlands

**Elleke Landeweer**

Netherlands

**Adele Langlois**

UK

**Graeme Laurie**

UK

**Karen Le Ball**

UK

**Brian Lee**

USA

**Aaron Levine**

USA

**Penney Lewis**

UK

**Charles Lidz**

USA

**Graham Lindegger**

South Africa

**Bebe Loff**

Australia

**Geir Lorem**

Norway

**Sana Loue**

USA

**Michael Loughlin**

UK

**Pekka Louhiala**

Finland

**Anneke Lucassen**

UK

**Andreas Lundh**

Denmark

**Jeroen Luyten**

UK

**Alan Lyles**

USA

**Niels Lynöe**

Sweden

**Malcolm Macleod**

UK

**Morten Magelssen**

Norway

**Barry Main**

UK

**Kathryn Maitland**

UK

**Mario Malicki**

Croatia

**Phillipa Malpas**

New Zealand

**Manassé Bambonyé**

Burundi

**Vicki Marsh**

Kenya

**Patricia Marshall**

USA

**Wayne Martin**

UK

**Fred Martineau**

UK

**Zubin Master**

USA

**Keikantse Matlhagela**

Botswana

**Doreen Matsui**

Canada

**Pauline McCormack**

UK

**Fiona McDermott**

Australia

**Alex McKeown**

UK

**Marcel Mertz**

Germany

**Kenneth Miller**

USA

**Bert Molewijk**

Netherlands

**Keymanthri Moodley**

South Africa

**Marilyn Morris**

USA

**Solange Nappo**

Brazil

**Darcia Narvaez**

USA

**Oktawian Nawrot**

Poland

**Joseph Ochieng**

Uganda

**Gearoid O'Cuinn**

UK

**Anke Oerlemans**

UK

**Dermot O'Reilly**

UK

**Dominik Ose**

Germany

**Gareth Owen**

UK

**John Owens**

UK

**Michael Parker**

UK

**Sarah Parsons**

UK

**Andy Petros**

UK

**Bernhard Pommer**

Austria

**Rouven Porz**

Switzerland

**Muireann Quigley**

UK

**Leah Rand**

UK

**Samiran Ray**

UK

**Sabi Redwood**

UK

**Dean Regier**

Canada

**Massimo Reichlin**

Italy

**Stuart Rennie**

USA

**David Resnik**

USA

**Solina Richter**

Canada

**Blanca Rodriguez Lopez**

Spain

**David Rodriguez-Arias**

Spain

**Wolf Rogowski**

Germany

**Philip Rosoff**

USA

**Michael Rowe**

USA

**Sabine Salloch**

Germany

**Lars Sandman**

Sweden

**Silke Schicktanz**

Germany

**Jan Schildmann**

Germany

**Sebastian Schleidgen**

Germany

**Harald Schmidt**

USA

**Guy Schofield**

UK

**Mark Schweda**

Germany

**Donnie Self**

USA

**Daniel Sessler**

USA

**Jane Seymour**

UK

**Anne Slowther**

UK

**Jeremy Snyder**

Canada

**Margaret Somerville**

Canada

**Antonio G Spagnolo**

Italy

**Dominique Sprumont**

Switzerland

**Shalini Sri Ranganathan**

Sri Lanka

**Ruth Stirton**

UK

**Lynne Stobbart**

UK

**John Stone**

USA

**Tuija Takala**

Finland

**Kate Tan**

Australia

**Attila Tanyi**

UK

**James Thomas**

USA

**Paulina Tindana**

Ghana

**Manuel Trachsel**

Switzerland

**Krista Tromp**

Netherlands

**Mark Tschaepe**

USA

**Edith Valdez-Martinez**

Mexico

**Rieke van der Graaf**

Netherlands

**Robert Van Howe**

USA

**Werdie van Staden**

South Africa

**Ghislaine van Thiel**

Netherlands

**Liliana Varesco**

Italy

**Dorothy Vawter**

USA

**Joseph Verheijde**

USA

**Giuseppe Vertrugno**

Italy

**Alice Virani**

Canada

**Polychronis Voultsos**

Greece

**Katherine Wade**

Ireland

**Julia Wade**

UK

**Elizabeth Wager**

UK

**Susan E Wallace**

UK

**Nancy Walton**

Canada

**Douglas Wassenaar**

South Africa

**Michael Weaver**

USA

**Guy Widdershoven**

Netherlands

**Claudia Wiesemann**

Germany

**Verina Wild**

Switzerland

**Stephen Wilkinson**

UK

**Garrath Williams**

UK

**Eva Winkler**

Germany

**Jean Woo**

Hong Kong

**Julie Woodley**

UK

**Simon Woods**

UK

**Bridget Young**

UK

**Matjaz Zwitter**

Slovenia

